# Depressive Symptoms among Latino Sexual Minority Men and Latina Transgender Women in a New Settlement State: The Role of Perceived Discrimination

**DOI:** 10.1155/2016/4972854

**Published:** 2016-09-14

**Authors:** Christina J. Sun, Alice Ma, Amanda E. Tanner, Lilli Mann, Beth A. Reboussin, Manuel Garcia, Jorge Alonzo, Scott D. Rhodes

**Affiliations:** ^1^OHSU-PSU School of Public Health, P.O. Box 751, SCH, Portland, OR 97207-0751, USA; ^2^University of North Carolina, Greensboro, P.O. Box 26170, Greensboro, NC 27402-6170, USA; ^3^Wake Forest School of Medicine, Medical Center Boulevard, Winston-Salem, NC 27157, USA

## Abstract

*Background.* Little is known about the role of discrimination on depression among Latino sexual and gender identity minorities. This manuscript examined the relationship between ethnic/racial discrimination and sexual discrimination on clinically significant depressive symptoms among Latino sexual minority men (i.e., gay and bisexual men and other men who have sex with men) and Latina transgender women.* Methods.* A community-based participatory research partnership recruited participants (*N* = 186; 80.6% cisgender men) in North Carolina to a social network-based HIV intervention. Using baseline data, we quantified the amount of perceived discrimination and conducted mixed-effects logistic regression analyses to examine correlates of clinically significant depressive symptoms.* Results.* A high percentage of participants reported ethnic/racial discrimination (73.7%) and sexual discrimination (53.8%). In the multivariable models, ethnic/racial discrimination, sexual discrimination, masculinity, fatalism, and social support were significantly associated with clinically significant depressive symptoms.* Discussion.* Improving mental health requires multilevel interventions that address pertinent individual, interpersonal, and system level factors.

## 1. Introduction

The Latino population is rapidly growing in the United States (US) and the South, particularly North Carolina (NC), has become an important new settlement region for immigrant Latinos [1]. Between 2000 and 2010, the Latino population increased by 57.3% in the South and more than double in NC [2]. Almost half of the Latino population in NC is foreign-born, with Mexico as the predominant country of origin [3]. Immigrant Latinos experience multiple barriers to health (e.g., language, isolation, and documentation status), which can increase the risk for negative health outcomes, including depression, and are exacerbated by a lack of bilingual and bicultural services in new settlement states [4].

The intersection of other identities (e.g., sexual and gender) can compound health risks [5]. Specifically, immigrant Latino sexual minorities may face an increased burden of health. Those who identify as sexual and gender identity minorities can include gay-identified persons, men who have sex with men (MSM) but who do not self-identify as gay, and gender variant/minority or transgender men (e.g., female-to-male transgender). Latino sexual minorities may be particularly vulnerable to mental health disparities. Among immigrant Latino sexual minority men residing in the South, US, Rhodes and colleagues [6] estimated the prevalence of clinically significant depressive symptoms to be between 69.2% and 74.8%.

Negative mental health outcomes can be affected by perceived discrimination, defined as a behavioral manifestation of negative attitudes, judgment, or unfair treatment toward members of a particular group [7]. Perceived discrimination, structural, institutional, and individual, can generate multiple stressors that adversely affect health [8]. For instance, discrimination is associated with psychological (e.g., emotional stress) and physiological (e.g., increased vulnerability to illness) distress [7, 9, 10]. The manifestation of depressive symptoms from discrimination-related stressors may be especially poignant for ethnic/racial and sexual minorities compared to their nonminority counterparts [11, 12]. Indeed, the burden of poor mental health associated with perceived ethnic/racial discrimination among Latinos may be further complicated by their sexual identity and orientation and gender identity [9, 13, 14].

Despite much extant literature on the association between perceived discrimination and mental health, limited research is available for sexual minority men and transgender women (exceptions [15–18]). The research that does exist suggests that, in general, perceived discrimination is associated with higher psychological distress (e.g., depression, anxiety, and suicidal ideation) [7, 9]. Existing studies have predominantly compared the experiences of Latinos in general to other races/ethnicities (e.g., African American/Black [19–21], in metropolitan areas [22–24], or in border states [25, 26]). Few studies have focused on the perceived discrimination experiences of immigrant Latino sexual minority men and Latina transgender women residing in the South; Latinos in new settlement states in the South may be differentially impacted by the lack of infrastructure to meet the unique needs and priorities of this emerging population (e.g., lack of bilingual and bicultural services, including mental health services) and the high levels of anti-immigration sentiment [3, 27–29]. Latinos living in the South, particularly if they are undocumented, may be concerned about the level of confidentiality and privacy available at mental healthcare facilities. If they perceive a lack of Latino-friendly services and recognize high anti-immigration sentiment in the community, they may be hesitant to access and engage in mental healthcare.

Given our focus on the experience of depression among Latino sexual and gender identity minorities, the conceptual framework for this study is the minority stress model [30–32]. This model posits that discrimination, stigma, and prejudice create a hostile and stressful environment that contribute to mental health issues. It identifies a variety of stress processes, including the experience of prejudice events, homophobia, and coping behaviors. This minority stress is particularly salient among sexual minorities compared to their heterosexual counterparts; in fact, the development of this model was motivated in part from observing this higher prevalence and seeking to understand it [32]. Based on the minority stress theory, sexual minority health disparities may be explained in part by a hostile, homophobic environment that then affects mental health [30]. We used the minority stress model to undergird our understanding of the relationship between perceived discrimination and depression among this population, as well as informing the development of our analysis. The purpose of this analysis was to examine the association between ethnic/racial discrimination and sexual identity or same-sex sexual behavior discrimination experienced by Latino sexual minority men and Latina transgender women on depression. Our specific aims were to quantify the amount of perceived discrimination based on ethnicity/race (hereafter ethnic/racial discrimination) and sexual identity or same-sex sexual behavior (hereafter sexual discrimination) experienced by Latino sexual minority men and Latina transgender women and then examine the association between reported discrimination and depression.

## 2. Methods

### 2.1. Participants and Data Collection

We examined HOLA intervention baseline survey data collected from November 2011 to July 2012 [33, 34]. Briefly, the HOLA intervention is a lay health advisor and social network intervention designed to increase HIV testing and condom use among immigrant adult Latino sexual minority men and Latina transgender women. Twenty-one individuals were recruited to serve as lay health advisors; each lay health advisor then recruited 8 Latino sexual minority men and Latina transgender women from his or her social network (*N* = 186). The intervention and evaluation plans were developed in response to community-identified needs and priorities by a community-based participatory research (CBPR) partnership, comprised of lay community members, organization representatives, and university health professionals and researchers, that has existed for more than 13 years [35]. The study protocol was approved by the Wake Forest School of Medicine Institutional Review Board.

### 2.2. Measures

#### 2.2.1. Demographic Characteristics

Participants reported their age, gender identity (female, male, male-to-female transgender, female-to-male transgender, and other), length of time they had lived in the US, highest level of education (dichotomized as less than a high school diploma or equivalent [GED] or at least a high school diploma or equivalent [GED]), employment status (dichotomized as employed year-round or not), and monthly income (dichotomized as less than $2,000 or at least $2,000).

#### 2.2.2. Alcohol Use

Participants reported the number of days they get drunk in a typical week. This single question has been shown to be an effective measure to assess alcohol use that is associated with higher risk for injury [36].

#### 2.2.3. Acculturation

Participants completed the Short Acculturation Scale for Hispanics [37]. This scale consists of three factors: language use, media, and ethnic social relations or socialization. For example, participants answered the question “What language(s) do you usually speak with your friends?” using the responses “Only Spanish,” “More Spanish than English,” “Both equally,” “More English than Spanish,” and “Only English.” The Cronbach's alpha for the entire scale in this sample was 0.87.

#### 2.2.4. Masculinity

Participants rated their agreement to items from the Conformity to Masculinity Norms Inventory [38], a scale developed to measure how strongly someone adhered to traditional masculine gender norms on a 4-point scale from “strongly disagree” (1) to “strongly agree” (4). While the CBPR partnership recognized and appreciated the psychometric properties and comprehensiveness of the original 144-item Conformity to Masculinity Norms Inventory that consists of 11 subscales, such as power over women, primacy of work, and disdain for homosexuals, the partnership was concerned about the participant burden. To reduce participant burden, members of the CBPR partnership reviewed all the items and decided to choose items that would be most salient and meaningful to the participants (e.g., Taking dangerous risks help me to prove myself). This process resulted in the selection of 26 items and was found to be reliable (Cronbach's alpha = 0.83).

#### 2.2.5. Internalized Homonegativity

Participants completed the shortened version of the Reactions to Homosexuality Scale [39]. Participants rated their agreement to 7 statements (e.g., Even if I could change my sexual orientation, I wouldn't.) on a 7-point scale from “strongly disagree” (1) to “strongly agree” (7). Cronbach's alpha was 0.83.

#### 2.2.6. Fatalism

Participants completed a previously validated and reliable fatalism scale [40]. This scale consists of three factors: predetermination, luck, and pessimism. Participants rated their agreement to 20 statements (e.g., If someone is meant to get a serious disease, it doesn't matter what they do, they will get that disease anyway.) on a 5-point scale from “strongly disagree” (0) to “strongly agree” (4). Cronbach's alpha was 0.92.

#### 2.2.7. Community Attachment

Participants rated their attachment to the gay community, Latino community, and Latino gay community on a 6-point scale from “not at all” (0) to “to a great extent” (5). The three items (e.g., Please indicate how much you feel a part of or connected to the Latino community.) were based on a previously used measure [41]. Cronbach's alpha was 0.88.

#### 2.2.8. Social Support

Participants completed the 18-item Index of Sojourner's Social Support [42, 43]. For each item, participants reported how many people would provide socioemotional support (e.g., comfort you whenever you feel homesick) and/or instrumental support (e.g., provide necessary information to help orient you to your new surroundings) on a 5-point scale from “no one would do this” (0) to “many people would do this” (4). Cronbach's alpha for the entire scale was 0.95.

#### 2.2.9. Discrimination

We measured ethnic/racial discrimination and sexual discrimination using a modified version of the Everyday Discrimination Scale [44]. This measure has been previously validated across ethnic/racial groups [45]. Participants reported in the past 12 months whether they had experienced 10 different types of discrimination (e.g., treated with less courtesy than other people) because of their race, ethnicity, or color (ethnic/racial discrimination) and because of their sexual identity or same-sex sexual behavior (sexual discrimination) with a “yes” or “no” response. We summed the number of “yes” responses such that higher scores reflect greater experiences of discrimination. In this sample, Cronbach's alpha for ethnic/racial discrimination was 0.83 and that for sexual discrimination was 0.81. The correlation between the discrimination measures was 0.71.

#### 2.2.10. Depressive Symptoms

Participants completed the widely used Center for Epidemiologic Studies Depression Scale (CES-D) [46]. Participants rated how often they experienced 20 symptoms (e.g., poor appetite, restless sleep, and feeling lonely) associated with depression during the past week on a 4-point scale (“rarely or none of the time, <1 day” [1], “some or a little of the time, 1-2 days” [2], “occasionally or a moderate amount of time, 3-4 days” [3], and “most or all of the time, 5–7 days” [4]). In accordance with standard scoring practice for the CES-D, participants who scored 16 or higher on the scale were classified as experiencing clinically significant depressive symptoms [47].

### 2.3. Analysis Plan

In the case of missing item-level data for a particular scale, if an individual was missing less than 20% of the items, person mean substitution was used; we replaced missing responses with that individual's mean response [48]. We calculated means, standard deviations, ranges, frequencies, and percentages to describe the sample. We performed bivariable mixed-effects logistic regression analyses to examine the relationship between demographic characteristics, psychosocial characteristics, behaviors, and perceived discrimination with clinically significant depressive symptoms. We then entered variables that were associated with clinically significant depressive symptoms (*p* < 0.25) into the multivariable mixed-effects logistic regression model to identify the independent contribution of each while adjusting for the effects of the other variables in the model; this is a standard approach to model building [49]. Because of the high correlation between ethnic/racial discrimination and sexual discrimination, two separate multivariable models were fit. As transgender women may differentially interpret the items for sexual discrimination (i.e., perceive themselves engaging in heterosexual behaviors), we conducted a subset analysis in which only cisgender men were included. All analyses were conducted in Stata version 12 and mixed-effects logistic regression analyses were conducted using the statistical procedure GLLAMM.

## 3. Results

### 3.1. Participant Characteristics

On average, participants were 30.1 years and had lived in the US for 10.1 years. Most of the sample identified as male (80.6%); 0.5% as female; 17.7% as male-to-female transgender; and 1.1% as* transvesti*. Nearly half (46.8%) had a high school diploma or equivalent. Nearly three-quarters (71.0%) were employed year-round; however, only one-fifth (19.9%) earned a monthly income of at least $2,000. Over one-third (37.6%) met the criterion for clinically significant depressive symptoms.  Table 1 summarizes participant characteristics.

Nearly three-quarters of the sample reported experiencing at least one type of ethnic/racial discrimination (73.7%) in the past 12 months and over half reported experiencing at least one type of sexual discrimination (53.8%) in the past 12 months. A higher percentage of participants reported ethnic/racial discrimination compared to sexual discrimination.

### 3.2. Correlates with Depressive Symptoms

The bivariable modeling shows age, employment status, masculinity, fatalism, and social support were significantly associated with clinically significant depressive symptoms (see Table 2). Both ethnic/racial discrimination and sexual discrimination were significantly associated with increased odds of clinically significant depressive symptoms. In the multivariable models, after adjusting for age, employment status, masculinity, fatalism, and social support, ethnic/racial discrimination and sexual discrimination were both significantly associated with increased odds of clinically significant depressive symptoms. Our additional analysis, with only cisgender men, showed that, after adjusting for factors associated with clinically significant depressive symptoms, sexual discrimination was marginally associated with increased odds of clinically significant depressive symptoms among Latino sexual minority men (AOR = 1.18, 95% CI = 0.98–1.42, *p* < 0.08).

## 4. Discussion

Overall, in this sample of Latino sexual minority men and Latina transgender women, we found high percentages of both ethnic/racial discrimination and sexual discrimination. Nearly three-quarters of participants (73.7%) reported experiencing ethnic/racial discrimination and over half reported experiencing sexual discrimination (53.8%). Further, over one-third (37.6%) of the participants met the cut-off criterion for clinically significant depressive symptoms. In addition to masculinity, fatalism, and social support significantly associated with clinically significant depressive symptoms, we found both ethnic/racial discrimination and sexual discrimination were associated with increased odds of clinically significant depressive symptoms. These results underscore the need to consider the role of different types of discrimination on mental health.

The rate of perceived ethnic/racial discrimination was higher among this sample compared to a national sample of Latinos; in the 2010 National Survey of Latinos, 34% of native-born and 33% of foreign-born Latinos reported that they or someone they know had experienced racial/ethnic discrimination in the past 5 years [50]. One potential reason for this difference is the particular context of the South and NC which includes a recent rapid growth in Latino populations, a lack of bilingual and bicultural infrastructure and resources, and greater hostility toward Latinos compared to states with longer histories of Latino immigration [3, 27–29].

The difference in experiences of perceived ethnic/racial discrimination and sexual discrimination may be understood by Goffman's [51] conceptualization of stigma. Stigma can be generated by more visible characteristics, such as ethnicity/race, or less visible characteristics, such as sexual identity; this has implications for what characteristics can be hidden and when “passing” is possible, possibly reducing experiences of discrimination. For Latino sexual minority men and Latina transgender women, passing as heterosexual or cisgender may minimize some discrimination, thereby, reducing multiple marginalization that may incur from embodying multiple minority statuses. When navigating mental and other healthcare services, the implications of passing can vary. On one hand, passing may be a coping strategy that decreases direct discrimination experiences; on the other hand, it may serve to hide or conceal one's identity, which can lead to stress and depression [32].

The significant association between both ethnic/racial discrimination and sexual discrimination and clinically significant depressive symptoms are similar to other studies [52]. When the analysis of the association between sexual discrimination and clinically significant depressive symptoms was limited to a subsample of cisgender men, the magnitude and direction of the association remained similar, but the statistical significance was reduced. Sexual discrimination scores between Latino sexual minority men and Latina transgender women were not significantly different (results not shown). Taken together, this suggests that, by limiting the sample size to 150, we may have reduced statistical power.

These results have several implications. Health practitioners should be aware of how discrimination is associated with clinically significant depressive symptoms and the need for culturally congruent mental health services for Latinos. A dearth of mental health services exists in new Latino settlement states like NC [53]. This is particularly problematic given that Latinos face many health access barriers, including mistrust, and thus may avoid seeking services that do exist [54]; this is especially true for Latino sexual minority men and Latina transgender women [55]. Our findings reinforce that more bilingual and bicultural mental health resources are needed that are accessible and consider factors such as discrimination, masculinity, fatalism, and social support. Thus, multilevel interventions should address pertinent individual, interpersonal, and system level factors that impact the care-seeking behaviors of multimarginalized populations.

Multilevel interventions to reduce clinically significant depressive symptoms and increase access to mental health services may involve increasing social support and addressing masculinity and fatalism. As demonstrated in these results and other research, social support can be a protective factor to reduce the odds of depression [56, 57]; additional research about sexual and gender identity minorities' social networks may provide important information about sources of social support to inform interventions. These results also showed that masculinity and fatalism were associated with increased odds of clinically significant depressive symptoms and interventions that address both factors may be an important component to enhance coping strategies to promote mental health.

Several limitations should be taken into account. The results of this analysis are based on cross-sectional data and causal inferences are not possible. Prospective studies have found a similar association, where experience of discrimination at an earlier time point was positively associated with depression at a later time point and not vice versa [20, 58, 59]. Participants were asked to recall experiences of discrimination that occurred in the past 12 months, and accurate recall of events may have been challenged. This small sample consisted of Latino sexual minority men and Latina transgender women who were willing to participate in the HOLA intervention and findings may not be generalizable; however, whereas this was a relatively small sample of willing participants, this is also a hard-to-reach population. Future research should include qualitative research to further understand how and when Latino sexual minority men and Latina transgender women experience various types of discrimination and to identify other possible mechanisms to temper the association between discrimination and clinically significant depressive symptoms.

## 5. Conclusion

Latino sexual minority men and Latina transgender women living in the South, experience profound ethnic/racial discrimination and sexual discrimination that are linked with clinically significant levels of depressive symptoms. This study points to the importance of external factors, including discrimination and social support, on mental health outcomes for multimarginalized persons. Efforts to improve mental health require multilevel interventions that address pertinent individual (e.g., counseling services), interpersonal (e.g., social support groups), and system (e.g., accessibility of bilingual and bicultural mental healthcare) level factors and consider the particular context in which they navigate their care-seeking behaviors. Addressing these factors can enhance access to mental health and other services and reduce the mental health burden for marginalized populations, such as Latino sexual and gender identity minorities.

## Figures and Tables

**Table 1:** Participant characteristics.

	M ± SD (range) or *n* (%)
Age (years)	30.1 ± 7.2 (18–61)
Length of time living in the US (years)	10.1 ± 5.4 (0.3–26.6)
≥HS diploma or equivalent	87 (46.8)
Employed year-round	132 (71.0)
≥$2,000 monthly income	37 (19.9)
Alcohol use (days/week)	0.8 ± 0.9 (0–4)
Acculturation	2.1 ± 0.7 (1–4.5)
Masculinity	52.3 ± 9.0 (30–78)
Internalized homonegativity	36.4 ± 9.1 (12–49)
Fatalism	44.1 ± 15.0 (20–88.1)
Community attachment	12.0 ± 4.3 (3–18)
Social support	55.9 ± 17.1 (18–90)
Ethnic/racial discrimination	3.0 ± 2.7 (0–10)
Sexual discrimination	2.0 ± 2.5 (0–8)
Clinically significant depressive symptoms (CES-D^a^ ≥ 16)	70 (37.6)

^a^CES-D = Center for Epidemiologic Studies Depression Scale.

**Table 2:** Bivariable and multivariable associations with clinically significant depressive symptoms.

	OR (95% CI)	AOR (95% CI)^a,b^	AOR (95% CI)^a,c^
Discrimination			
Ethnic/racial	1.28 (1.13, 1.45)^†^	1.24 (1.05, 1.46)^†^	—
Sexual identity or same-sex sexual behavior	1.24 (1.09, 1.41)^†^	—	1.22 (1.03, 1.44)^†^
Control variables			
Age (years)	0.97 (0.92, 1.01)^‡^	0.99 (0.93, 1.04)	0.99 (0.93, 1.05)
Employment status (year-round)	0.54 (0.26, 1.11)^‡^	1.56 (0.56, 4.36)	1.49 (0.54, 4.11)
Masculinity	1.03 (0.99, 1.07)^‡^	1.04 (0.99, 1.09)^‡^	1.04 (0.99, 1.08)^‡^
Fatalism	1.03 (1.00, 1.05)^‡^	1.03 (1.00, 1.06)^‡^	1.03 (1.00, 1.06)^‡^
Social support	0.98 (0.96, 1.00)^‡^	0.98 (0.96, 1.01)^‡^	0.98 (0.96, 1.01)^‡^
Other sociodemographics			
Length of time lived in the US (years)	0.98 (0.92, 1.04)	—	—
≥HS diploma or equivalent	0.73 (0.37, 1.43)	—	—
≥$2,000 monthly income	0.76 (0.34, 1.74)	—	—
Alcohol use (days/week)	0.80 (0.53, 1.20)	—	—
Acculturation	0.82 (0.50, 1.33)	—	—
Internalized homonegativity	0.99 (0.95, 1.03)	—	—
Community attachment	0.98 (0.91, 1.06)	—	—

^‡^ *p* < 0.25 and  ^†^ *p* < 0.05. ^a^Adjusted for age, employment status, masculinity, fatalism, and social support. ^b^Model to examine the association between ethnic/racial discrimination and clinically significant depressive symptoms. ^c^Model to examine the association between sexual discrimination and clinically significant depressive symptoms.
